# Semi-rational design and engineering of chicory-derived germacrene A synthase for enhanced (−)-β-elemene production in *Saccharomyces cerevisiae*

**DOI:** 10.1186/s12896-026-01212-3

**Published:** 2026-08-12

**Authors:** Da-Ming Wang, Si-Jia Song, Kang-Yue Ren, Lei Sun, Yong-Kun Du, Hao Guo, Tian Xie

**Affiliations:** 1 Key Laboratory of Elemene Class Anti-Cancer Medicines, School of Pharmacy, Hangzhou Normal University, Hangzhou, 311121 P. R. China; 2 School of Food and Biological Engineering, Jiangsu University, Zhenjiang, 212013 P. R. China

**Keywords:** Germacrene A synthase, β-elemene, Site-directed mutagenesis, *Saccharomyces cerevisiae*

## Abstract

In this study, we applied a semi-rational engineering strategy to improve the catalytic performance and operational stability of germacrene A synthase from *Cichorium intybus* (CiGASlo), a key biocatalyst for stereochemically defined (–)-β-elemene biosynthesis. An AlphaFold2-predicted model was first validated by confidence-score analysis, stereochemical-quality assessment, and structural comparison with a related plant germacrene A synthase. Molecular docking was then used to define substrate-proximal residues around farnesyl pyrophosphate (FPP), while alanine scanning and HotSpot Wizard 3.0 were used as stability- and mutational-tolerance filters rather than as direct predictors of catalytic activity. Experimental screening of a focused mutation library identified several variants with improved activity, including D337H, L415W, D477H, E485Y, R488A, W305A, and the double variant W305A/L415W. Experimental screening identified W305A/L415W as the variant with the highest specific activity under the fixed assay conditions (0.95 ± 0.03 U·mg^− 1^; 69.6% above wild type). Subsequent characterization showed that L415W, rather than the double variant, had the more favorable *K*m, *k*cat/*K*m, and numerical thermal profile. Expression of W305A/L415W in the engineered yeast strain produced 5.44 g·L^− 1^ (−)-β-elemene under continuous feeding, demonstrating feasibility of this strain configuration without establishing superiority over an L415W-containing strain. This work provides a combined enzyme-engineering and fermentation-validation strategy for improving plant terpene synthases in microbial cell factories.

**Graphical Abstract:**

**Figure:**
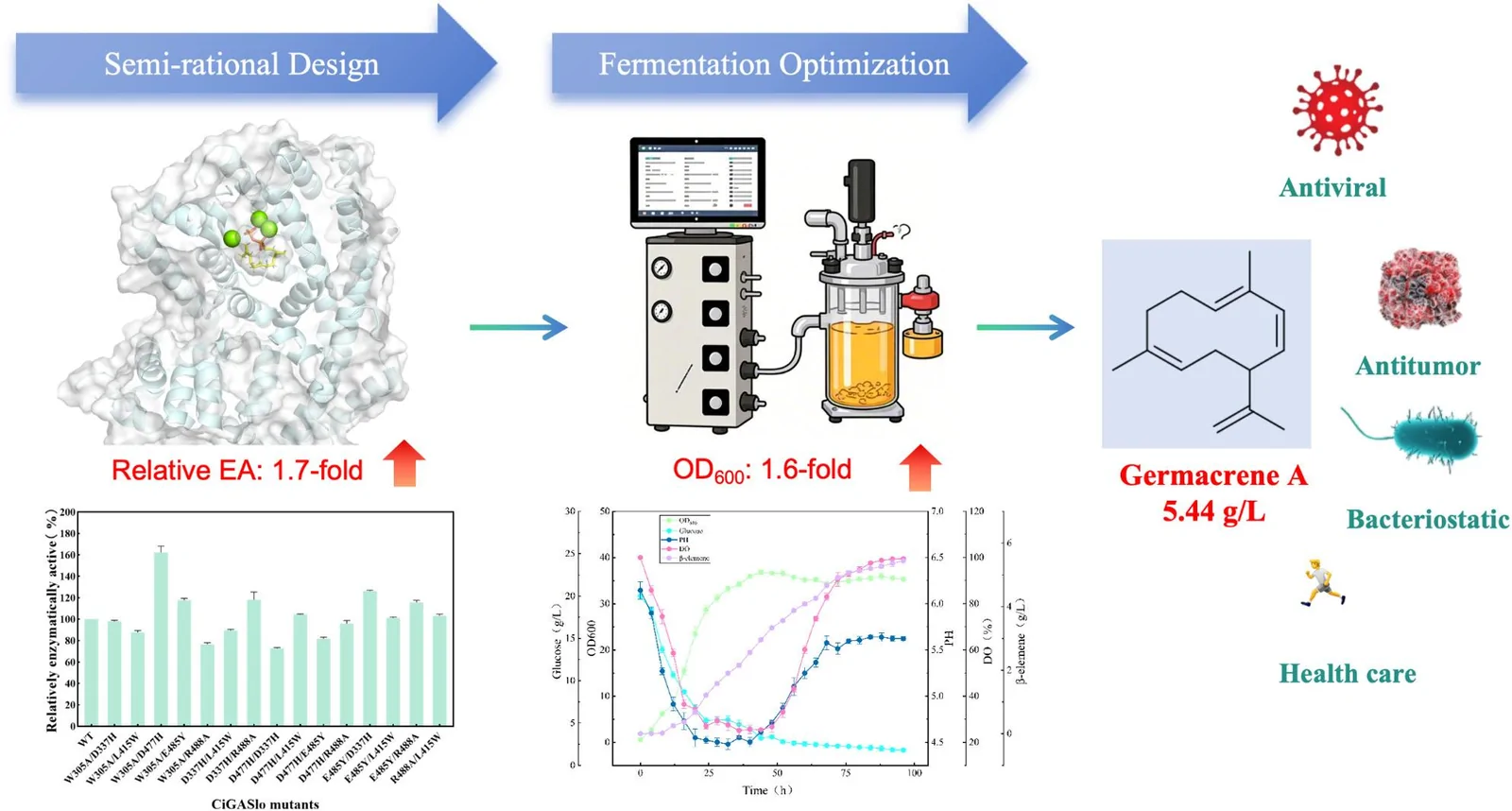

**Supplementary Information:**

The online version contains supplementary material available at https://doi.org/10.1186/s12896-026-01212-3.

## Introduction

β-Elemene, a bioactive sesquiterpene originally derived from Curcuma wenyujin, is a clinically validated broad-spectrum antitumor agent [3, 8, 38, 40]. It has been widely investigated or applied in the treatment of lung [29, 35], ovarian [25], gastric [16, 30], and breast cancers [8, 9, 31, 37, 40]. Its antitumor efficacy is associated with multiple mechanisms, including inhibition of tumor proliferation, induction of apoptosis, suppression of metastasis, and modulation of drug-resistance-related signaling pathways. Traditionally, β-elemene is obtained by extraction from plant materials [44] or by chemical synthesis [4, 5, 11]. However, plant extraction is limited by low natural abundance, slow growth cycles, and environmental variability, whereas chemical synthesis often requires harsh conditions or toxic catalysts and may generate undesired stereoisomers. These limitations have stimulated increasing interest in sustainable microbial biosynthesis using synthetic biology and metabolic-engineering strategies [18, 24, 26, 27].

Microbial production of β-elemene depends on the efficient supply and stereochemically controlled conversion of farnesyl pyrophosphate (FPP), the universal C15 precursor generated through the mevalonate (MVA) or methylerythritol phosphate pathway [2]. Germacrene A synthase (GAS) catalyzes the Mg^2+^-dependent cyclization of FPP to germacrene A, a chemically unstable intermediate that can undergo spontaneous Cope rearrangement to form β-elemene [14, 42]. Therefore, efficient microbial β-elemene biosynthesis requires not only enhanced FPP flux but also a GAS enzyme capable of directing FPP folding toward the stereochemically appropriate germacrene A intermediate. Recent metabolic-engineering efforts have substantially improved precursor supply and host performance in *Escherichia coli*, *Saccharomyces cerevisiae*, *Ogataea polymorpha*, and *Yarrowia lipolytica* (Table S1). For example, heterologous reconstruction of the MVA pathway in *E. coli* and *S. cerevisiae*, together with suppression of competing pathways, increased β-elemene production to 3.52 g·L^-1^ in fed-batch fermentation [17]. Nonconventional hosts have further expanded production capacity, with *O. polymorpha* producing 4.7 g·L^-1^ β-elemene and *Y. lipolytica* producing 39 g·L^-1^ germacrene A through systematic rewiring of central carbon metabolism and NADPH/ATP supply [28, 43]. Despite these advances, GAS performance remains a major bottleneck because catalytic efficiency, operational stability, and stereochemical control collectively determine the final productivity and product configuration of β-elemene biosynthesis.

GASs are the gatekeeping enzymes that convert FPP into germacrene A and thereby determine both catalytic flux and product stereochemical outcome. Structural and mechanistic studies have shown that terpene synthases control product formation by shaping FPP conformation, carbocation stabilization, and cyclization trajectories within their active pockets [13, 20]. Plant-derived GASs tend to orient FPP toward (+)-germacrene A formation, which is relevant to the production of pharmaceutically active (−)-β-elemene, whereas some microbial homologs may generate incompatible stereochemical products or follow divergent folding patterns [7, 21]. For example, GASs from *Lactuca sativa*, *Cichorium intybus*, *cyanobacteria*, and other plant or microbial sources have been used for germacrene A or β-elemene biosynthesis, but their catalytic efficiencies and product profiles differ markedly (Table S2). LsLTC2 from *Lactuca sativa* exhibits a *k*cat/*K*m of 0.026 s^-1^·µM^-1^ [32]. By comparison, the recently characterized LcTPS3 from *Liriodendron chinense* shows a higher catalytic efficiency, with a *k*cat/*K*m of 0.107 s^-1^·µM^-1^ [12]. At the production level, LcTPS3 enabled 14.71 g·L^-1^ germacrene A production in yeast [12]. In another host system, global metabolic rewiring of *Ogataea polymorpha* enabled 4.7 g·L^-1^ β-elemene production [43]. These findings indicate that both GAS catalytic performance and host metabolic capacity jointly determine microbial β-elemene production.

Structural analysis of LcTPS3 further revealed that residue A280 participates in substrate cyclization by modulating FPP folding, while Y282L redirects product formation toward α-guaiene, highlighting the functional plasticity of GAS active sites [12]. However, the mechanistic basis underlying GAS stereochemical control remains incompletely understood, and the lack of experimentally solved structures for many GAS enzymes continues to limit structure-guided engineering [41]. Mutational scanning has therefore become an effective strategy for systematically identifying functionally important residues and exploring sequence–function relationships [23]. Fusion-protein engineering has also been widely applied to improve substrate channeling and catalytic efficiency in terpene biosynthesis [19]. More recently, computationally assisted residue prioritization and AI-guided protein engineering have provided increasingly efficient approaches for rational enzyme optimization [10, 39]. Nevertheless, semi-rational engineering, which combines computational prediction with experimental validation, remains one of the most practical and effective strategies for improving GAS catalytic performance [6].

CiGASlo from *Cichorium intybus* is an attractive engineering target because it originates from a plant source associated with (+)-germacrene A biosynthesis and is therefore relevant to stereochemically defined β-elemene production. Compared with microbial GAS homologs that may generate divergent stereochemical products, plant-derived GASs provide more suitable starting points for producing the desired β-elemene enantiomer. In addition, BLAST search, multiple-sequence alignment, and phylogenetic analysis showed that CiGASlo retains the conserved class I terpene synthase motifs, including the DDXXD and NSE/DTE motifs required for Mg^2+^-dependent diphosphate activation, and clusters within the plant TPS-a clade. These features, together with its promising substrate affinity but suboptimal turnover and stability, make CiGASlo a suitable candidate for semi-rational engineering.

In the present study, we focused on the engineering of CiGASlo to improve its catalytic and operational performance for β-elemene biosynthesis. We combined AlphaFold2-based structural modeling, molecular docking, mutational-tolerance prediction, site-directed mutagenesis, kinetic characterization, stability evaluation, and MD-assisted structural interpretation to identify CiGASlo variants with improved activity or stability. The selected variant was further introduced into *S. cerevisiae* to evaluate whether enzyme-level improvement could be translated into enhanced β-elemene production under bioreactor fermentation conditions. This work provides a focused enzyme-engineering strategy for improving plant-derived GASs and supports the development of microbial cell factories for stereochemically reliable β-elemene biosynthesis.

## Materials and methods

### Plasmid, strains and chemicals

The recombinant plasmid pET28a-CiGASlo harboring the *Cichorium intybus* germacrene A synthase gene (CiGASlo, NCBI accession No. AAM21658) was previously constructed and preserved in our laboratory. *Escherichia coli* DH5α was used as the host for gene cloning, and *E. coli* BL21(DE3) was used for gene expression.

Plasmids used in this study included pET28a(+) and pET28a-CiGASlo. KOD One™ PCR Master Mix was purchased from TOYOBO (Japan), and Dpn I restriction enzyme was obtained from Takara (Japan). The Bradford protein assay kit was purchased from Beyotime (China). Primers were synthesized by Tsingke Biotechnology Co., Ltd. (Beijing, China). β-D-thiogalactopyranoside (IPTG) and kanamycin were purchased from Solarbio (China). Yeast extract and peptone were obtained from OXOID (UK), and FPP was purchased from Sigma (USA).

### Protein expression and purification

For protein expression, a single confirmed recombinant colony was inoculated into 10 mL of LB medium supplemented with 50 µg/mL kanamycin and cultured at 37 °C, 180 rpm for 10 h. Then, 1% (v/v) of the seed culture was transferred into 1 L of fresh LB medium containing kanamycin and incubated at 37 °C until the optical density at 600 nm (OD_600_) reached 0.6 ~ 0.8. Protein expression was induced by adding IPTG to a final concentration of 0.1 mM. The culture was then incubated at 17 °C with shaking at 180 rpm for 20 h. Cells were harvested by centrifugation at 8000 rpm for 10 min using a floor centrifuge.

The collected cell pellets were resuspended in lysis buffer (50 mM Tris-HCl, pH 7.4, 150 mM NaCl, 20 mM imidazole, 0.1% (v/v) Tween 20, and 1 mM PMSF) and disrupted using a high-pressure homogenizer at 1000 ~ 1200 bar and 4 °C. After three passes, the lysate was centrifuged at 9000 rpm for 45 min to remove cell debris. The resulting supernatant was subjected to Ni^2+^-affinity chromatography for purification of the His-tagged recombinant protein. Protein concentration was determined using a BCA protein assay kit, and protein purity and molecular weight were analyzed by SDS-PAGE. All protein purification steps were conducted at 4 °C.

### Site-directed mutagenesis

Site-directed mutagenesis was performed using inverse PCR with the plasmid pET-28a(+)-CiGASlo as the template. Primers used for each mutation are listed in Table S3. PCR amplification was carried out with the following cycling conditions: initial denaturation at 98 °C for 5 min; followed by 30 cycles of denaturation at 98 °C for 30 s, annealing at 55 °C for 30 s, and extension at 72 °C for 90 s; with a final extension step at 72 °C for 5 min. The PCR products were then treated with Dpn I to digest the parental methylated DNA template. The resulting plasmids were transformed into *E. coli* BL21(DE3) competent cells and plated on LB agar plates containing 50 µg/mL kanamycin. After incubation at 37 °C for 15 h, colonies were screened and verified by DNA sequencing to confirm the presence of the desired mutations.

### Structural modeling, model validation, and molecular docking of CiGASlo

The three-dimensional structure of CiGASlo was predicted de novo using AlphaFold2. The amino acid sequence of CiGASlo was retrieved from the NCBI database and submitted for structure prediction [22]. The resulting model was evaluated before molecular docking.

First, the per-residue confidence score (pLDDT) was analyzed to assess the reliability of the predicted structure. Regions with high pLDDT values were considered reliable for active-site and catalytic-core analysis, whereas low-confidence terminal or flexible regions were not used for mechanistic interpretation. Second, the stereochemical quality of the model was evaluated using Ramachandran plot analysis and model-quality assessment tools, including PROCHECK, ERRAT, Verify3D, or MolProbity [1]. Third, the predicted CiGASlo structure was compared with the experimentally solved structure of a related plant germacrene A synthase from *Nicotiana tabacum* (PDB: 5EAU). The spatial conservation of the DDXXD and NSE/DTE motifs was used to evaluate whether the predicted active-site architecture was suitable for docking and residue-prioritization analysis.

Before docking, the CiGASlo model was prepared using the Protein Preparation Wizard module in the Schrödinger Suite [15, 33]. Missing side chains and loops were repaired where necessary, hydrogen atoms were added, protonation states were assigned at pH 7.0 ± 0.5, the hydrogen-bonding network was optimized, and restrained energy minimization was performed using the OPLS_2005 force field. The substrate FPP and Mg^2+^ ions were prepared using LigPrep. The docking grid was centered on the conserved catalytic motifs and the putative FPP-binding pocket. The grid box was set to cover the active-site cavity and adjacent substrate-access region. Docking was performed using Glide with standard precision followed by extra precision docking for the top-ranked poses. The final binding pose was selected based on GlideScore, coordination of the Mg^2+^ cluster, conservation of catalytic-motif geometry, and visual inspection of substrate orientation. The AlphaFold2 model was used as a structural guide for semi-rational mutagenesis and hypothesis generation, but not as a substitute for experimentally solved high-resolution structures.

### Residue prioritization and virtual mutagenesis

A focused semi-rational mutagenesis strategy was used to reduce the experimental library size while retaining residues most likely to influence substrate positioning, catalytic-site architecture, or local stability. Residues located within 4 Å of the docked FPP molecule were defined as first-shell substrate-proximal residues. This distance cutoff was selected because residues within approximately 4 Å can directly contribute to van der Waals contacts, hydrogen bonding, electrostatic interactions, steric shaping of the pocket, or metal/substrate positioning. The 4 Å cutoff was therefore used as a practical criterion for identifying residues forming the immediate substrate-binding microenvironment.

It should be noted that predicted ΔΔG values obtained from alanine scanning or HotSpot Wizard analysis were interpreted as predicted changes in folding stability or mutational tolerance, not as substrate-binding free energy or direct indicators of catalytic activity. Molecular docking was used to identify substrate-proximal residues, whereas ΔΔG-based prediction was used only as a secondary filter to identify structurally tolerated or potentially stabilizing substitutions. Final evaluation of catalytic improvement was based on experimental enzyme activity, kinetic parameters, and stability assays.

The mutation workflow consisted of four steps. First, docking analysis was used to identify FPP-proximal residues in the CiGASlo active pocket. Second, alanine scanning was performed on selected residues to evaluate whether removal of side-chain bulk or specific side-chain interactions affected activity. Third, HotSpot Wizard 3.0 was used to suggest additional substitutions predicted to be structurally tolerated or stabilizing. Fourth, experimentally beneficial single variants were selected for pairwise combination. Full saturation mutagenesis of all 20 amino acid substitutions at every candidate site was not performed; instead, a focused library was constructed to balance rational design with experimental feasibility.

**Fig. 1:**
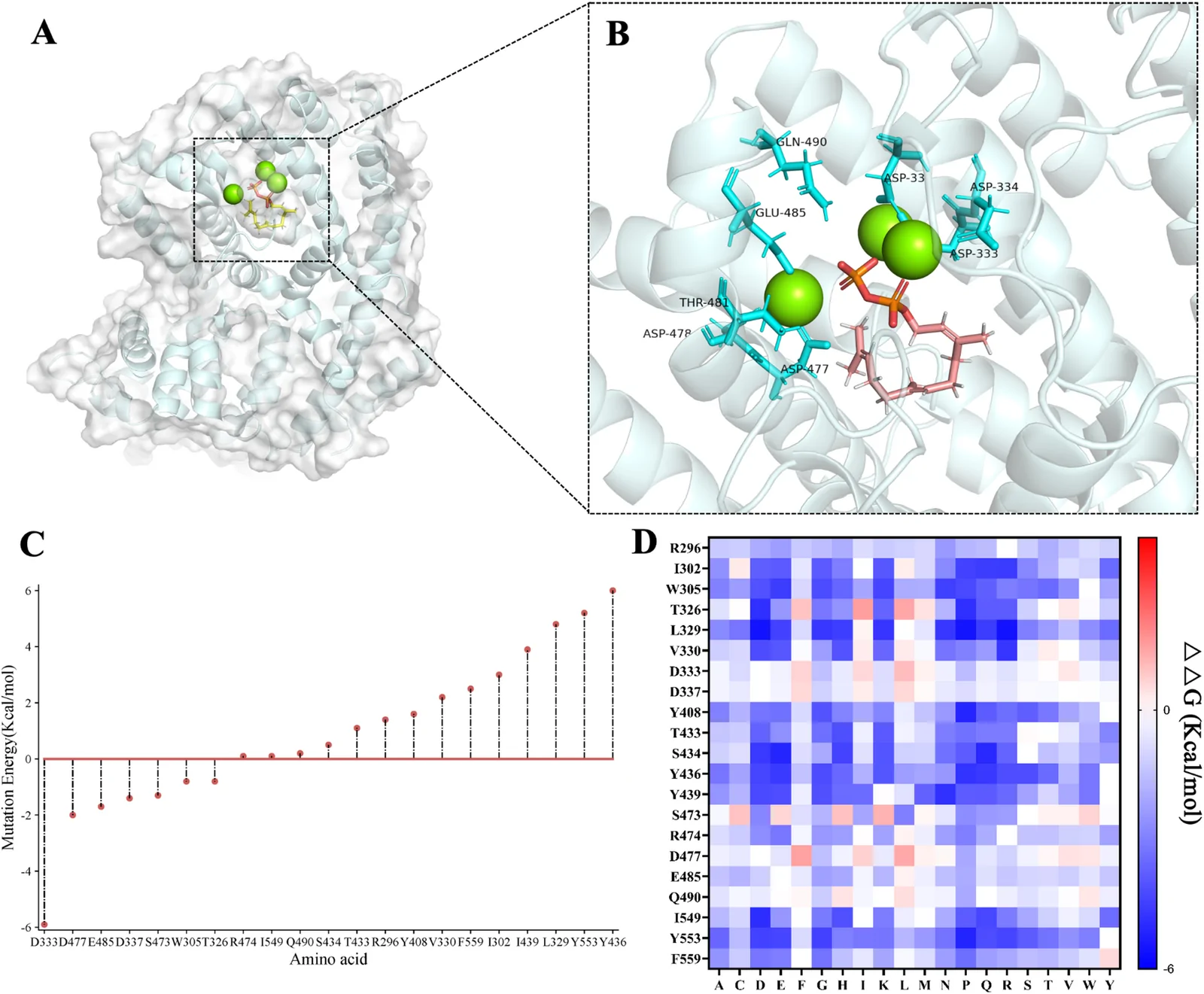
Structural analysis and residue prioritization of CiGASlo. **A**, Docking model of FPP in the predicted CiGASlo active pocket and substrate-proximal residues identified within 4 Å of FPP. **B**, Close-up view of the catalytic pocket showing the FPP orientation, Mg^2+^ ions, and conserved catalytic motifs. **C**, Predicted folding-stability changes from alanine scanning of selected substrate-proximal residues. ΔΔG values were used as a mutational-tolerance and stability-oriented filter, not as direct binding free energy. **D**, Activity screening of selected single-site variants generated from alanine scanning and HotSpot Wizard-guided substitutions

After rechecking the docking-derived residue list, the inconsistency between the 22 docking-identified residues and the final 21 mutation sites in the original manuscript was corrected. The revised manuscript now explicitly states that D478 was retained in the final candidate list, and the final residue list was made consistent across the main text, Fig. 1, and Table S4.

### Enzyme activity assay of CiGASlo and its variants

Germacrene A synthase activity was assayed using FPP as the substrate. The standard reaction mixture contained 10 mM MgCl_2_, 50 mM Tris-HCl buffer (pH 7.4), 20 mM β-mercaptoethanol, 10% (v/v) glycerol, 0.1% (v/v) Tween 20, purified recombinant enzyme, and FPP in a final aqueous volume of 0.5 mL. After gentle mixing, the aqueous reaction mixture was overlaid with 0.5 mL n-hexane. The hexane layer served as a hydrophobic extraction phase to capture volatile terpene products and reduce product loss, rather than as the catalytic medium. Because n-hexane has very low miscibility with water, the enzyme remained predominantly in the aqueous phase during the reaction.

The reaction was incubated at 30 °C for 30 min. Vigorous mixing was applied only after the enzymatic reaction to facilitate extraction of the product into the hexane phase before GC analysis. The mixture was then centrifuged at 10,000 × g for 5 min, and the upper hexane layer was collected for GC analysis. Control assays confirmed that the short-term biphasic extraction condition did not cause substantial loss of enzyme activity under the selected assay conditions. One unit of enzyme activity was defined as the amount of enzyme required to produce 1 µmol of germacrene A per minute under the assay conditions. Specific activity was calculated as units per milligram of purified enzyme. All activity measurements were performed in triplicate.

### Enzymatic characterization of CiGASlo and its variants

The optimum temperature was determined by measuring enzyme activity at 20, 25, 30, 35, 40, 50, and 60 °C under otherwise identical assay conditions. The highest activity observed in this temperature range was defined as 100%, and relative activity was calculated accordingly.

Thermal stability was evaluated by pre-incubating purified enzymes at 20, 30, 40, 50, and 60 °C for up to 3 h. Samples were withdrawn at defined time intervals, cooled on ice, and assayed under standard conditions. Residual activity was calculated by defining the activity before heat treatment as 100%. All thermal-stability data were rechecked to ensure that residual activity followed a physically reasonable trend and that any small differences within experimental error were not overinterpreted.

The optimum pH was determined using citrate buffer, phosphate buffer, and Tris-HCl buffer covering pH 4.0–9.0. For pH-stability analysis, purified enzymes were incubated in buffers of different pH values at 30 °C for 24 h and then assayed under standard conditions. Residual activity was calculated relative to the activity of the untreated control.

For kinetic analysis, initial reaction rates were measured at FPP concentrations ranging from 0.5 to 100 µM. Product formation was maintained within the linear range with respect to reaction time and enzyme concentration. For each of three independent experiments, the initial-rate data were fitted separately to the Michaelis–Menten equation by nonlinear regression to obtain *K*m and *V*max. The *k*cat value was calculated from the fitted Vmax and the molar concentration of purified enzyme, and catalytic efficiency was calculated as *k*cat/*K*m. The kinetic parameters obtained from the three independent fits were subsequently summarized as mean ± SD.

**Fig. 2:**
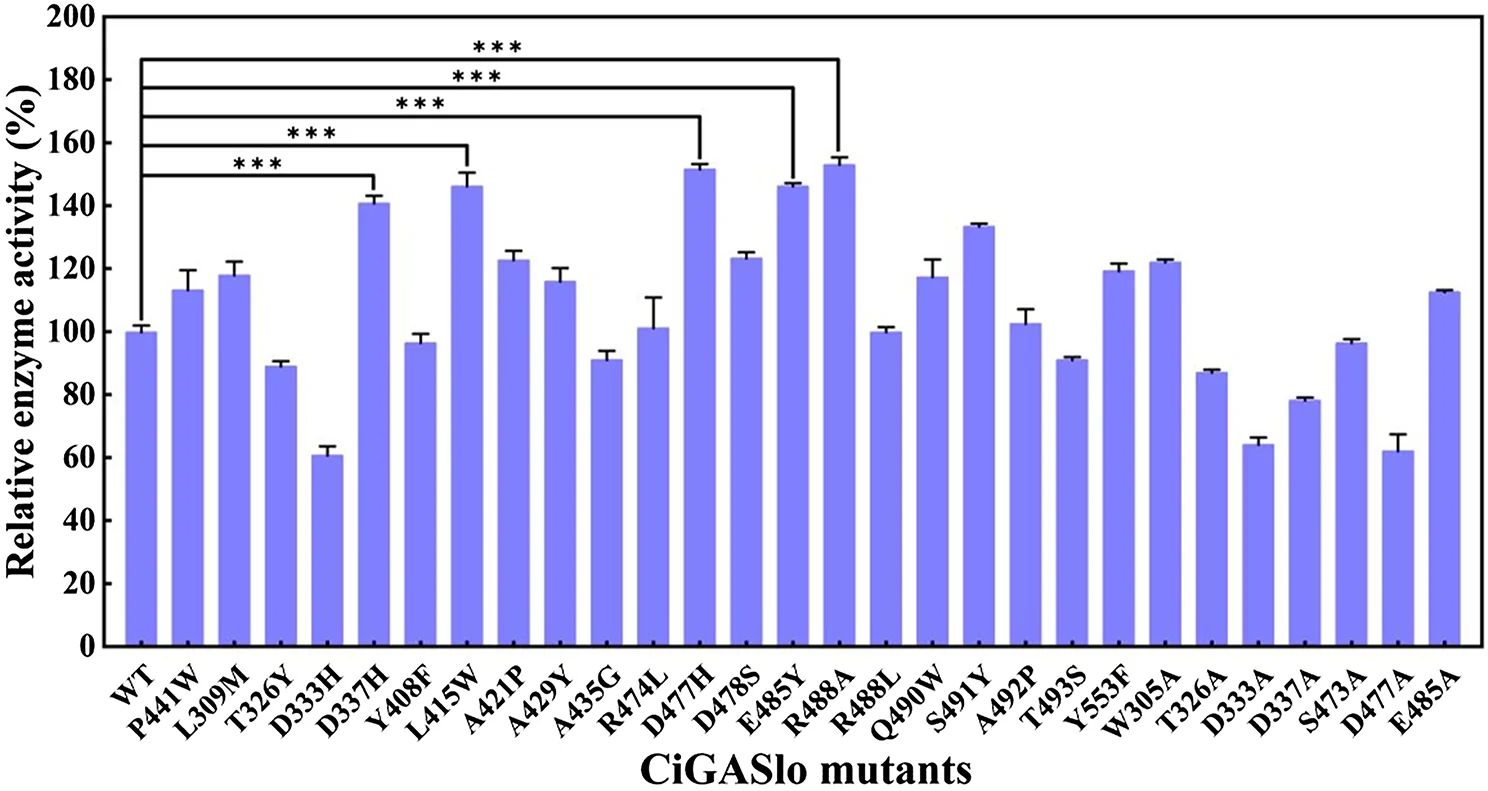
Relative enzyme activities of CiGASlo single-site variants. The activity of wild-type CiGASlo under the standard assay conditions (0.56 ± 0.02 U·mg^− 1^) was defined as 100%. Data are presented as mean ± SD from three independent experiments. Statistical significance for each variant versus wild-type CiGASlo was determined using one-way ANOVA followed by Dunnett’s multiple-comparison test. Only significant comparisons are indicated: ***p < 0.001. L415W showed significantly higher activity than the wild type (***p < 0.001)

**Fig. 3:**
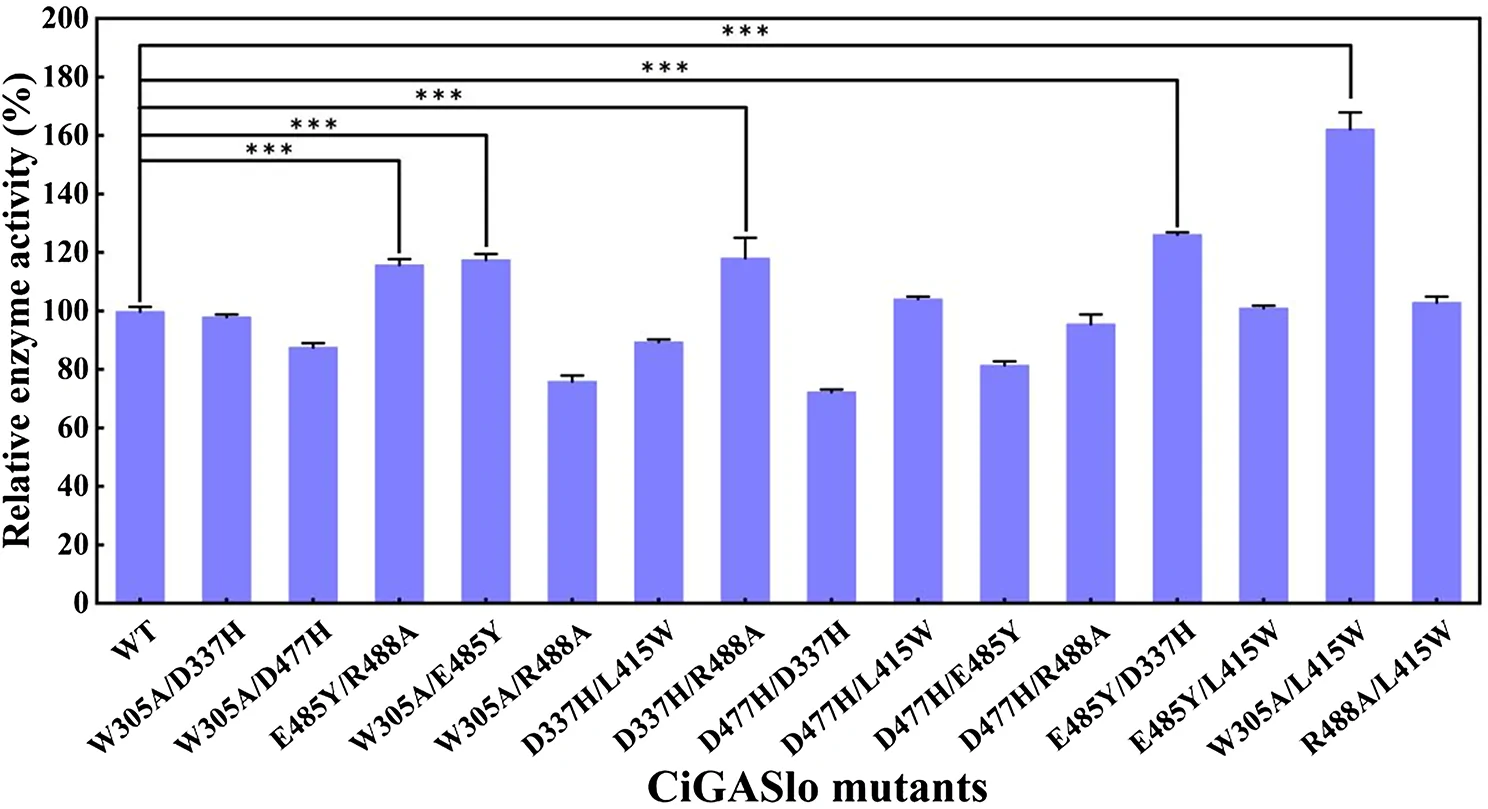
Relative enzyme activities of CiGASlo double-site variants. The activity of wild-type CiGASlo under the standard assay conditions (0.56 ± 0.02 U·mg^− 1^) was defined as 100%. Data are presented as mean ± SD from three independent experiments. Statistical significance for each double variant versus wild-type CiGASlo was determined using one-way ANOVA followed by Dunnett’s multiple-comparison test. Only significant comparisons are indicated: ***p < 0.001

**Fig. 4:**
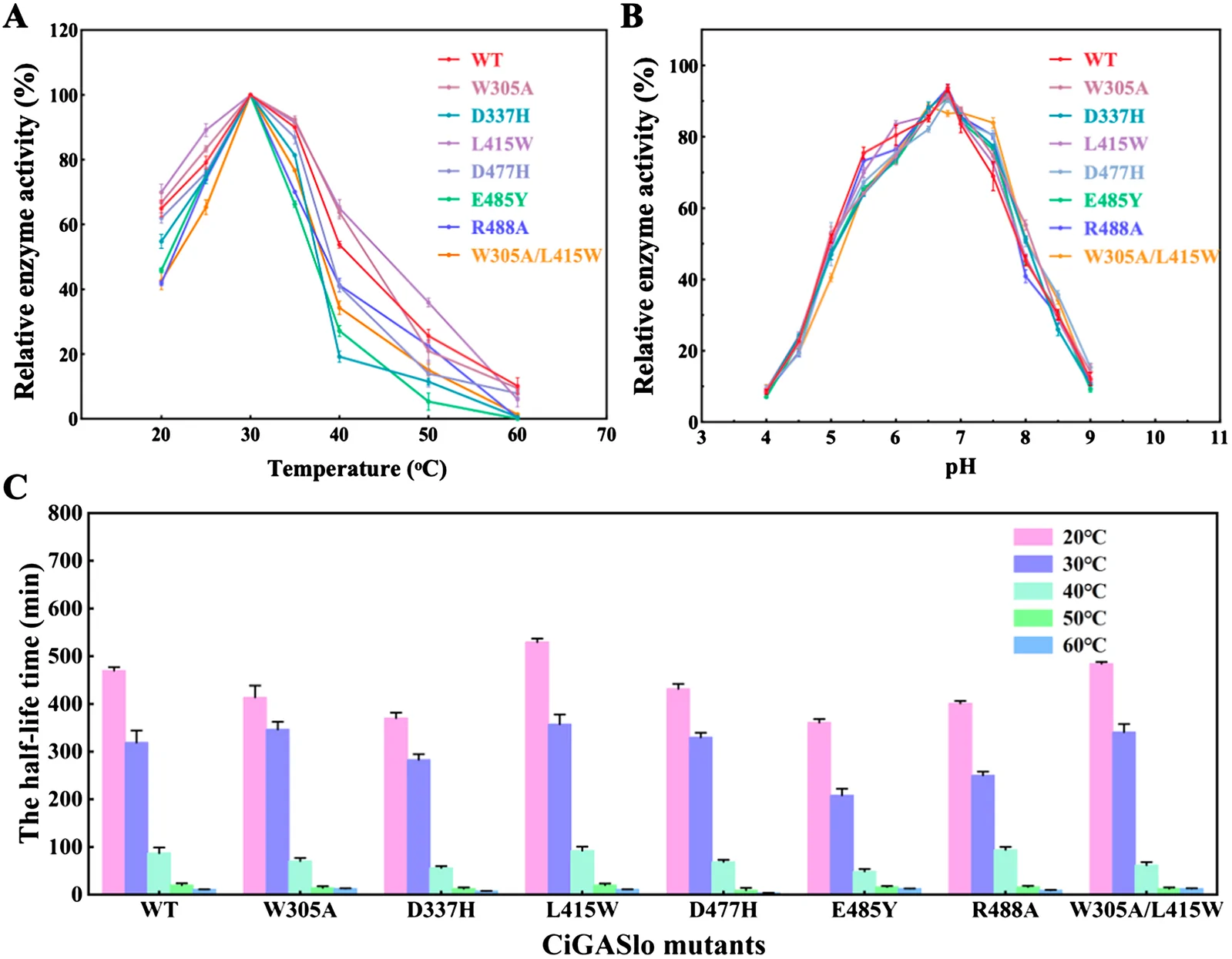
Biochemical characterization of wild-type CiGASlo and selected variants. **(A)** Temperature–activity profiles measured from 20 to 60 °C. For each enzyme, its activity at 30 °C was defined as 100%. **(B)** pH-stability profiles after incubation for 24 h at 30 °C. For each enzyme, the activity measured before pH incubation under the standard assay conditions was defined as 100%, with the corresponding absolute activities given above. **(C)** Thermal half-lives at 20, 30, 40, 50, and 60 °C, determined independently for each replicate by fitting the time-dependent residual-activity data to a one-phase exponential-decay model. Bars represent mean ± SD from three independent experiments

Unless otherwise stated, all quantitative experiments were performed in three independent replicates, and the results are presented as mean ± standard deviation (SD). For Figs. 2 and 3, differences between each CiGASlo variant and the wild-type enzyme were evaluated using one-way analysis of variance (ANOVA) followed by Dunnett’s multiple-comparison test, with wild-type CiGASlo serving as the control. Thermal half-lives in Fig. 4C were determined independently for each replicate by fitting the residual-activity data to a one-phase exponential-decay model. The resulting half-life values were subsequently summarized as mean ± SD. Statistical significance was defined as follows: ns, not significant; *p < 0.05; **p < 0.01; ***p < 0.001; and ****p < 0.0001.

### Construction of recombinant strain

In this study, the GAL1 promoter, known for its strong inducibility, and the CYC1 terminator were selected to drive the expression of the CiGAS1o gene in *Saccharomyces cerevisiae*. To ensure stable genomic integration without disrupting native gene functions, the neutral site NSXV-3 (chromosome XV: 459,184~460,418) was chosen as the integration locus. The expression cassette CiGAS1o-GAL1-CYC1 was constructed and targeted to the NSXV-3 site. A specific single-guide RNA (sgRNA) plasmid, gRNA-NSXV-3, was designed using overlapping PCR techniques to facilitate CRISPR/Cas9-mediated integration. The accuracy of the sgRNA construct was confirmed through DNA sequencing.

The expression cassette, along with the gRNA-NSXV-3 and a Cas9-expressing plasmid, were co-transformed into a previously engineered S. cerevisiae BY4742 strain via electroporation. Transformants were selected on YPD agar plates supplemented with ampicillin and incubated at 30 °C for 72 h. Single colonies were picked and cultured in 5 mL of YPD liquid medium containing ampicillin at 30 °C with shaking at 220 rpm for 12 ~ 16 h until the optical density at 600 nm (OD600) reached 0.8 ~ 1.0. Subsequently, 2% (v/v) of this culture was inoculated into 250 mL of YPD medium with ampicillin and grown under the same conditions until OD600 reached 6 ~ 7, serving as the seed culture for subsequent fermentation processes. The resulting recombinant strain was designated as B2-11.

### Fermentation strategies

To optimize β-elemene production, three fermentation strategies were evaluated using 5 L bioreactors, each conducted in quadruplicate: batch fermentation, fed-batch fermentation, and continuous feeding fermentation. The fermentation were conducted at 30 °C and pH control at 6.5-7.0, with agitation rate between 200 and 400 rpm, DO value at 10%–30%. Batch Fermentation: The fermentation was carried out for 72 h under standard conditions. Samples (5 mL) were collected every 4 h to measure β-elemene concentration and residual glucose levels. Fed-Batch Fermentation: Building upon the batch fermentation protocol, 60 g of glucose was added at the 12-hour mark, followed by an additional 60 g of glucose and 30 g of yeast extract at 24 h. Continuous Feeding Fermentation: Starting at 12 h into the batch fermentation, yeast extract and glucose were continuously fed into the bioreactor at rates of 2 g·L-1·h-1 and 4 g·L-1·h-1, respectively, over a 24-hour period.

The fermentation experiments in this study were designed to validate whether the engineered CiGASlo variant could improve β-elemene accumulation in a yeast cell-factory context. Therefore, three representative feeding strategies, including batch, fed-batch, and continuous feeding fermentation, were compared. A full statistical Design of Experiment (DoE) was not performed at this stage. However, the current fermentation results provide a basis for future DoE-guided optimization of key process variables, including glucose feeding rate, yeast extract feeding rate, pH, dissolved oxygen, agitation speed, and fermentation temperature.

### Molecular dynamics simulation and quantitative structural analysis

Molecular dynamics simulations were performed to compare the conformational behavior of wild-type CiGASlo and selected variants. The starting structures were prepared from the validated structural models. Each system was placed in a cubic simulation box with TIP3P water molecules, neutralized with appropriate counterions, and subjected to energy minimization. The systems were then equilibrated under NVT and NPT ensembles before production MD simulation.

Trajectory analysis included root mean square deviation (RMSD), root mean square fluctuation (RMSF), radius of gyration (*R*g), intramolecular hydrogen-bond occupancy, and local structural analysis around the active-site and substrate-access regions. *R*g values were reported as mean ± standard deviation over the equilibrated portion of the trajectory rather than as single-point values. Hydrogen-bond analysis focused on occupancy and persistence of local interactions rather than only the total number of hydrogen bonds. Where applicable, substrate-pocket or channel properties, including pocket volume, bottleneck radius, or solvent-accessible surface area, were quantified using appropriate structural-analysis tools. MD simulations were interpreted as supportive structural analyses for generating mechanistic hypotheses, not as definitive proof of catalytic mechanism [34, 36]. The system was equilibrated in two phases: first under a constant number of particles, volume, and temperature (NVT ensemble), followed by a constant number of particles, pressure, and temperature (NPT ensemble), to ensure thermodynamic and pressure stability, respectively. After equilibration, production MD simulations were conducted at 303 K for 100 ns with a time step of 2 fs.

## Results and discussion

### Structural validation and identification of candidate mutation sites

CiGASlo was selected as the engineering target based on literature evidence, BLAST analysis, and multiple-sequence alignment. As a plant-derived germacrene A synthase from *Cichorium intybus*, CiGASlo is mechanistically relevant to the stereochemically appropriate biosynthesis of germacrene A, which can subsequently undergo Cope rearrangement to form the desired β-elemene enantiomer. Sequence alignment showed that CiGASlo retains the conserved class I terpene synthase motifs, including the DDXXD and NSE/DTE motifs required for Mg^2+^-dependent activation of farnesyl pyrophosphate (FPP). Phylogenetic analysis further indicated that CiGASlo clusters with plant TPS-a-type sesquiterpene synthases rather than microbial GAS homologs, supporting its suitability as a starting enzyme for stereochemically defined β-elemene biosynthesis.

Before molecular docking and mutagenesis design, the AlphaFold2-predicted CiGASlo structure was evaluated to confirm its suitability for active-site analysis. The catalytic-core region containing the conserved metal-binding motifs showed high structural confidence, whereas lower-confidence regions were mainly located in terminal or flexible loop segments. To further validate the predicted model, the CiGASlo structure was compared with the X-ray structure of Nicotiana tabacum germacrene A synthase (PDB: 5EAU) (Figure S2). The conserved catalytic motifs were spatially aligned between the two structures, supporting the use of the AlphaFold2 model for substrate-pocket mapping and semi-rational mutagenesis. Additional stereochemical-quality evaluation by Ramachandran plot and model-quality assessment also indicated that the predicted model was acceptable for guiding residue prioritization, although experimental structural determination will still be required for definitive mechanistic validation.

Molecular docking with FPP was then performed to map the substrate-binding pocket and identify residues forming the first-shell substrate microenvironment (Figure S3). A 4 Å cutoff was used because residues within this distance are generally capable of directly shaping substrate binding, substrate folding, metal coordination, or product-release pathways through van der Waals contacts, electrostatic interactions, hydrogen bonding, or steric effects. Docking identified 22 residues located within 4 Å of FPP or lining the binding pocket: R296, I302, W305, T326, L329, V330, D333, D337, Y408, T433, S434, Y436, I439, R474, S473, D477, D478, E485, Q490, I549, Y553, and F559 (Fig. 1A). Three Mg^2+^ ions were positioned near the conserved D333-D334-T-Y-D337 motif on helix D and the D477-D478-X-X-T-X-X-F-E485 motif on helix H, consistent with the typical Mg^2+^-dependent activation mechanism of class I terpene synthases. Specifically, the D333-D334-T-Y-D337 motif coordinates Mg^2+^_A_ and Mg^2+^_C_, whereas the D477-D478-X-X-T-X-X-F-E485 motif contributes to Mg^2+^_B_ coordination. In the docked complex, the pyrophosphate moiety of FPP forms a salt bridge with Arg296, while the hydrophobic tail of FPP adopts a U-shaped conformation stabilized by aromatic interactions with Trp305 and Phe559 (Fig. 1B).

To rationally guide CiGASlo engineering, we implemented a stepwise computational and experimental prioritization strategy. First, the 22 substrate-proximal residues identified by docking were used as the initial active-pocket residue pool. In silico alanine scanning was then performed to evaluate the predicted mutational tolerance of these residues. Importantly, the ΔΔG values obtained from alanine scanning were interpreted as predicted folding-stability or mutational-tolerance effects, not as substrate-binding free energy or direct predictors of catalytic activity. Among the 22 residues, seven residues—W305, T326, D333, D337, S473, D477, and E485—showed favorable predicted stability changes upon alanine substitution (ΔΔG ≤ − 0.5 kcal/mol) and were therefore selected for experimental alanine mutagenesis (Fig. 1C).

Second, to identify additional residues that may influence local stability or active-site architecture beyond the first alanine-scanning subset, the AlphaFold2-predicted CiGASlo structure was submitted to HotSpot Wizard 3.0. This analysis predicted 19 active-site-proximal residues with potentially favorable mutational effects: L309, T326, D333, D337, Y408, L415, A421, N429, A435, R474, D477, D478, E485, R488, Q490, S491, A492, T493, and Y553 (Table S4). These residues were considered complementary to the docking-derived alanine-scanning candidates because they may affect local packing, stability, or the dynamic environment of the catalytic pocket.

Finally, the seven alanine-scanning candidates and the 19 HotSpot Wizard-prioritized residues were merged, and duplicate entries were removed. This yielded a final set of 21 non-redundant mutation sites: W305, T326, D333, D337, S473, D477, E485, L309, Y408, L415, A421, N429, A435, R474, D478, R488, Q490, S491, A492, T493, and Y553. Thus, the apparent difference between the 22 docking-identified residues and the final 21 mutation sites reflects two different selection levels: the former represents all residues within the 4 Å substrate-binding environment, whereas the latter represents the merged and filtered set of residues selected for focused mutagenesis. These 21 sites were subsequently prioritized for single-point mutagenesis and experimental functional validation.

### Single-point mutagenesis of CiGASlo

Based on the substrate-proximal residue analysis and mutational-tolerance prediction described above, a focused single-site mutation library was constructed to experimentally evaluate the contribution of selected active-pocket residues to CiGASlo activity. This library was not designed as exhaustive saturation mutagenesis of all 20 amino acid substitutions at every candidate position. Instead, a stepwise semi-rational strategy was adopted. Alanine substitutions were first introduced at selected substrate-proximal residues to probe the effects of removing side-chain bulk, local steric constraints, or residue-specific interactions. In parallel, additional substitutions suggested by HotSpot Wizard 3.0 were introduced at residues predicted to tolerate mutation or potentially improve local folding stability. Therefore, the final set of single variants represented a focused, computation-guided library rather than a random or fully saturated mutation library.

For the alanine-scanning step, mutations were introduced at residues located within or adjacent to the FPP-binding pocket. Enzyme assays showed that W305A exhibited a 22% increase in relative activity compared with the wild-type enzyme (Fig. 1C), suggesting that the bulky indole side chain at this position may partially restrict substrate access or product release. Replacement of Trp305 with alanine may reduce steric hindrance near the pocket entrance and provide a more favorable local environment for catalysis. In contrast, S473A and E485A showed no significant difference from the wild type, whereas the remaining alanine substitutions led to reduced activity. In particular, D333A and D337A, which are located in the conserved D333-D334-T-Y-D337 motif on helix D, decreased catalytic activity despite their favorable predicted folding-stability changes. This result indicates that these acidic residues are important for maintaining the Mg^2+^-dependent catalytic geometry. Similarly, D477A led to diminished product formation in vitro, consistent with the role of D477 in the helix H metal-binding motif. These results demonstrate that predicted mutational tolerance or stability alone is insufficient to infer catalytic benefit, especially for residues involved in Mg^2+^coordination.

To complement alanine scanning, the AlphaFold2-predicted CiGASlo structure was analyzed using HotSpot Wizard 3.0 to identify additional active-site-proximal positions and candidate substitutions that may influence local stability or catalytic-pocket architecture. Seven positions were prioritized based on favorable predicted folding-stability changes (ΔΔG ≤ − 0.5 kcal/mol) and structural proximity to the catalytic pocket. The corresponding variants were constructed, expressed, purified, and assayed under identical conditions. Heat-map analysis of relative activities revealed five variants with pronounced activity improvements (Fig. 1D): D337H (+ 42%), L415W (+ 46%), D477H (+ 52%), E485Y (+ 46%), and R488A (+ 53%). Other variants showed more modest activity increases ranging from 1% to 33% (Fig. 2).

Activity screening identified five single-site variants—D337H, L415W, D477H, E485Y, and R488A—with significantly higher relative activities than wild-type CiGASlo (Fig. 2). Their relative activities were approximately 141%, 146%, 152%, 146%, and 153%, respectively. W305A also showed a reproducible numerical increase in activity and was therefore retained, together with these five statistically significant variants, for subsequent kinetic, stability, and combinatorial-mutagenesis analyses. These results demonstrate that the focused mutagenesis strategy identified several substitutions capable of enhancing CiGASlo activity, although the magnitude and statistical significance of the effects varied among positions.

### Combinatorial mutagenesis of CiGASlo

Based on the single-site screening results, six substitutions—W305A, D337H, L415W, D477H, E485Y, and R488A—were selected for pairwise combination, generating 15 double-site variants. Eight double variants showed activities numerically higher than that of wild-type CiGASlo, and five of these increases reached statistical significance according to one-way ANOVA followed by Dunnett’s multiple-comparison test (Fig. 3). Among the double variants, W305A/L415W showed the highest specific activity under the fixed screening conditions, reaching 0.95 ± 0.03 U·mg^− 1^ compared with 0.56 ± 0.02 U·mg^− 1^ for the wild type, corresponding to a 69.6% increase.

Nevertheless, the improved fixed-condition specific activity of W305A/L415W should not be interpreted as a uniformly synergistic improvement in all catalytic properties. Subsequent kinetic and thermal characterization revealed a trade-off: the double variant exhibited higher specific activity, Vmax, and kcat than L415W, whereas L415W retained a lower Km, higher kcat/Km, and a more favorable numerical thermal-stability profile. Thus, combining W305A with L415W redistributed the performance advantages across different catalytic and stability metrics rather than producing an additive improvement in every property.

### Enzymatic characterization of advantageous variants of CiGASlo

To elucidate how the selected mutations affected the catalytic properties, substrate response, and stability of CiGASlo, the six advantageous single variants W305A, D337H, L415W, D477H, E485Y, and R488A, together with the double variant W305A/L415W, were expressed in *E. coli*, purified to near homogeneity as confirmed by SDS-PAGE (Figure S4), and characterized in parallel with the wild-type enzyme under identical assay conditions. Apparent specific activity, temperature-dependent activity, thermal stability, pH stability, and apparent kinetic parameters were determined using FPP as the substrate.

Temperature-dependent activity assays performed from 20 °C to 60 °C showed that wild-type CiGASlo and all tested variants shared a common optimum temperature of approximately 30 °C (Fig. 4A). Enzyme activity increased from 20 °C to 30 °C, but declined rapidly when the reaction temperature exceeded 30 °C, indicating that CiGASlo was thermolabile under the tested assay conditions. Thermal-stability assays were then performed by pre-incubating purified enzymes at 20 °C, 30 °C, 40 °C, 50 °C, and 60 °C for up to 3 h, followed by residual-activity measurement under the standard assay condition. Thermal stability was further quantified from time-dependent inactivation experiments by independently fitting the residual-activity data from each replicate to a one-phase exponential-decay model. The resulting thermal half-lives decreased markedly with increasing temperature for all enzymes (Fig. 4C). L415W showed the most favorable numerical thermal profile, displaying longer half-lives than W305A/L415W at 20, 30, and 40 °C. At 40 °C, for example, the half-lives of L415W and W305A/L415W were approximately 95 and 65 min, respectively. At 50 and 60 °C, all enzymes were rapidly inactivated and exhibited comparatively short half-lives. These results indicate that introducing W305A into the L415W background did not improve the thermal robustness of L415W, despite increasing its fixed-condition specific activity and turnover rate.

The pH stability of wild-type CiGASlo and its variants was evaluated after incubation at different pH values for 24 h at 30 °C (Fig. 4B). The wild-type enzyme retained approximately 80% of its activity near neutral pH, but activity decreased markedly outside the pH 6.5–7.5 range. Several single variants, particularly E485Y, D337H, and R488A, showed higher residual activity than the wild type at mildly acidic or mildly alkaline pH values, suggesting improved tolerance to pH fluctuation. E485Y maintained relatively high residual activity over a broader pH range, indicating that this substitution may contribute to local structural stabilization under non-optimal pH conditions. By contrast, W305A/L415W showed lower long-term pH stability, with residual activity dropping below 60% even near the optimal pH after 24 h incubation. Therefore, W305A/L415W was not the most pH-robust variant, although it showed favorable thermal residual activity around 30 °C. These results indicate that different mutations improved distinct enzyme properties, and that catalytic activity, thermal stability, and pH robustness are not necessarily coupled.

Apparent kinetic parameters were determined from initial-rate measurements obtained over an FPP concentration range of 0.5–100 µM (Table 1; Figure S5). For each independent experiment, the initial-rate data were fitted separately to the Michaelis–Menten equation by nonlinear regression, yielding independent estimates of *K*m and Vmax. The resulting *K*m and Vmax values are therefore reported as mean ± SD from three independent fits. The corresponding *k*cat and *k*cat/*K*m values were calculated separately for each replicate and subsequently summarized as mean ± SD. The specific activity measured at 20 µM FPP is reported independently as a fixed-condition activity-screening metric and was not used to estimate Vmax.

**Table 1:** Kinetic parameters of wild-type CiGASlo and its variants

Variants	Apparent specific activity at 20 µM FPP (U·mg^− 1^)	Km (µM)	Vmax (U·mg^− 1^)	kcat (min^− 1^)	kcat/Km (M^− 1^·min^− 1^)	Fold vs. WT
CiGASlo wild-type	0.56 ± 0.02	10.52 ± 1.05	0.86 ± 0.04	55.55 ± 2.75	5.28 × 10^6^	1.00
CiGASlo-W305A	0.66 ± 0.02	1.81 ± 0.07	0.72 ± 0.02	46.78 ± 1.43	2.59 × 10^7^	4.90
CiGASlo-D337H	0.79 ± 0.03	8.87 ± 0.17	1.14 ± 0.04	74.12 ± 2.85	8.36 × 10^6^	1.58
CiGASlo-L415W	0.82 ± 0.03	2.36 ± 0.32	0.92 ± 0.04	59.59 ± 2.34	2.53 × 10^7^	4.78
CiGASlo-D477H	0.85 ± 0.03	8.61 ± 0.81	1.22 ± 0.06	79.04 ± 3.58	9.18 × 10^6^	1.74
CiGASlo-E485Y	0.82 ± 0.02	5.57 ± 0.43	1.05 ± 0.03	68.14 ± 2.02	1.22 × 10^7^	2.32
CiGASlo-R488A	0.86 ± 0.03	1.52 ± 0.18	0.93 ± 0.03	60.15 ± 2.16	3.96 × 10^7^	7.49
CiGASlo-W305A/L415W	0.95 ± 0.03	6.29 ± 0.34	1.25 ± 0.04	81.17 ± 2.77	1.29 × 10^7^	2.44

Data are presented as mean ± SD from three independent experiments. Specific activity was measured under the standard assay conditions using 20 µM FPP. For each independent experiment, *K*m and Vmax were obtained by nonlinear regression fitting of the initial-rate data to the Michaelis–Menten equation. The corresponding kcat and *k*cat/*K*m values were calculated independently for each replicate before calculation of the mean and SD. Fold values were calculated from the mean *k*cat/*K*m relative to that of wild-type CiGASlo

The wild-type enzyme exhibited an apparent specific activity of 0.56 ± 0.02 U·mg^-1^, a *K*m value of 10.52 ± 1.05 µM, an apparent Vmax of 0.855 U·mg^-1^, a *k*cat of 55.55 min^-1^, and a *k*cat/*K*m value of 5.28 × 10^6^ M^-1^·min^-1^ (Table 1). Compared with the wild type, all selected variants showed higher apparent specific activities under the standard screening condition, ranging from 0.66 ± 0.02 U·mg^-1^ for W305A to 0.95 ± 0.03 U·mg^-1^ for W305A/L415W. These results confirmed that the focused mutagenesis strategy successfully identified variants with improved product-forming activity under the assay condition.

Although R488A exhibited the highest apparent catalytic efficiency among the tested variants, L415W showed a favorable combination of substrate affinity, catalytic efficiency, and thermal robustness. Relative to wild-type CiGASlo, the *K*m of L415W decreased from 10.52 ± 1.05 to 2.36 ± 0.32 µM, whereas its *k*cat/*K*m increased from 5.28 × 10^6^ to 2.53 × 10^7^ M^-1^·min^-1^, corresponding to a 4.78-fold improvement. Contrary to the previous description, the *k*cat of L415W (59.59 min^-1^) was slightly higher than that of the wild type (55.55 min^-1^). Adding W305A to the L415W background increased the fixed-condition specific activity from 0.82 ± 0.03 to 0.95 ± 0.03 U·mg^-1^, the Vmax from 0.92 to 1.25 U·mg^-1^, and the *k*cat from 59.59 to 81.17 min^-1^. However, the additional substitution increased *K*m from 2.36 ± 0.32 to 6.29 ± 0.34 µM and reduced *k*cat/*K*m from 2.53 × 10^7^ to 1.29 × 10^7^ M^-1^·min^-1^, representing a 49.0% decrease in catalytic efficiency relative to L415W. These results demonstrate that W305A and L415W do not combine additively across all performance dimensions; instead, their combination produces a trade-off between fixed-condition activity and turnover on the one hand and substrate affinity, catalytic efficiency, and thermal robustness on the other.

W305A/L415W displayed the highest fixed-condition specific activity (0.95 ± 0.03 U·mg^-1^), but it was not superior across all kinetic and thermal metrics. Its *k*cat/*K*m was 1.29 × 10^7^ M^-1^·min^-1^, corresponding to a 2.44-fold increase relative to wild-type CiGASlo but a 49.0% decrease relative to L415W. Compared with L415W, W305A/L415W showed a 15.9% higher specific activity and a 36.2% higher *k*cat, but a 2.67-fold higher *K*m and a less favorable numerical thermal profile. Introducing W305A into the L415W background therefore generated a property trade-off rather than a uniformly synergistic improvement.

Collectively, the apparent kinetic analysis revealed that the engineered variants improved CiGASlo performance through different mechanisms. R488A was the most efficient variant in vitro based on *k*cat/*K*m, primarily because of its markedly reduced *K*m. W305A and L415W also substantially improved apparent catalytic efficiency by lowering *K*m, although W305A showed a slight decrease in turnover. D477H and W305A/L415W showed higher apparent *V*max and *k*cat values, suggesting improved product-forming capacity, but their catalytic efficiencies were limited by higher *K*m values. Therefore, the selection of W305A/L415W for yeast fermentation was based not on the highest *k*cat/*K*m, but on its highest apparent specific activity, highest apparent *V*max, highest *k*cat, and favorable residual activity at 30 °C. Since in vivo β-elemene accumulation depends on cumulative product formation during long-term cultivation, this balanced activity–stability profile was considered more relevant for fermentation validation than purified-enzyme catalytic efficiency alone.

### MD simulation and structural analysis of advantageous variants

**Fig. 5:**
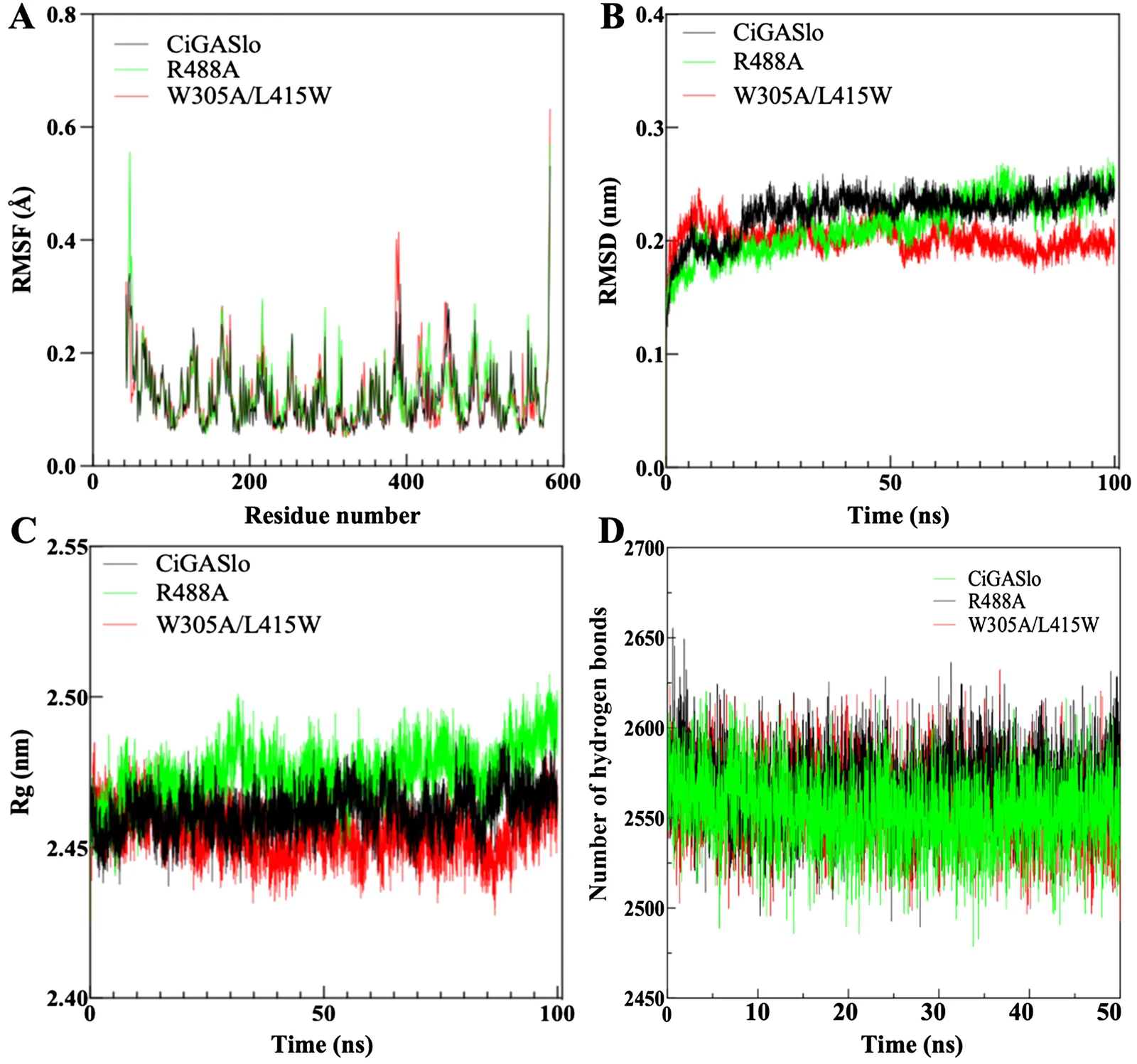
Molecular dynamics analysis of wild-type CiGASlo and selected variants. **A**, RMSD profiles showing overall trajectory stability. **B**, RMSF profiles showing residue-level flexibility. **C**, Radius of gyration reported as mean ± standard deviation over the equilibrated trajectory. **D**, Hydrogen-bond occupancy and/or persistent local interactions around the mutation sites. **E**, Quantitative pocket or channel descriptors of the substrate-access region, where applicable. MD simulations were used to provide supportive structural hypotheses and were not interpreted as definitive mechanistic proof

To further explore the possible structural basis underlying the altered catalytic and stability profiles of selected CiGASlo variants, molecular dynamics (MD) simulations were performed at 303 K for 100 ns. The wild-type enzyme and representative variants, including R488A, W305A/L415W, D333A, D337A, D477A, and E485A, were included in the simulation analysis. Because the simulations were based on predicted structural models, the MD results were interpreted as supportive structural hypotheses rather than definitive mechanistic validation. Trajectory analyses focused on overall stability, local flexibility, metal-coordination geometry, intramolecular hydrogen-bond patterns, and local structural changes around the substrate-binding pocket (Fig. 5).

The RMSD profiles indicated that all simulated systems reached relatively stable trajectories after equilibration, suggesting that no large-scale unfolding or global structural disruption occurred during the 50 ns simulations (Fig. 5A). RMSF analysis further showed that the mutations mainly affected local flexibility around active-site-adjacent regions and selected loop segments, rather than inducing major global conformational rearrangements (Fig. 5B). These observations are consistent with the experimental results showing that most variants retained catalytic activity, although their kinetic and stability profiles differed markedly.

Because germacrene A synthase catalysis depends on Mg^2+^-assisted FPP activation, the local geometry of the conserved metal-binding motifs was examined in detail. D333 and D337 are located in the conserved D333-D334-T-Y-D337 motif on helix D, which contributes to Mg^2+^_A_ and Mg^2+^_C_ coordination. The D333A and D337A substitutions reduced activity experimentally, and the simulations suggested that replacing these acidic residues with alanine weakened local metal-coordination interactions and perturbed the geometry of the catalytic center. Thus, although these substitutions may reduce local flexibility in some regions, the loss of key acidic side chains likely compromises the Mg^2+^-dependent organization required for efficient FPP ionization.

A similar trend was observed for residues located in the helix H metal-binding region. D477 is part of the D477-D478-X-X-T-X-X-F-E485 motif, which contributes to Mg^2+^_B_ coordination. The D477A mutation markedly decreased product formation, consistent with disruption of a critical metal-binding residue. In contrast, E485A had a less severe effect on catalytic activity, suggesting that the remaining coordinating residues may partly preserve the Mg^2+^-binding geometry. These results indicate that not all mutations within the metal-binding motif have equivalent functional consequences, and that the catalytic output depends on the balance between local stability and preservation of the metal-coordination network (Fig. 5C).

The R488A variant showed the highest apparent catalytic efficiency among the tested enzymes. RMSF analysis suggested altered local flexibility near the active-site exit region, including residues 579–585, which may influence substrate access or product release (Fig. 5B). However, the small change in global radius of gyration observed for R488A was not considered sufficient evidence for a global expansion of the enzyme or a definitive enlargement of the substrate entrance channel. Therefore, the possible role of R488A was interpreted more cautiously: replacement of Arg488 with alanine may locally reshape the electrostatic and steric environment near the pocket periphery, thereby facilitating a more favorable substrate-access or product-release process. This interpretation is consistent with the reduced Km and improved apparent catalytic efficiency of R488A, but further structural validation will be required.

**Fig. 6:**
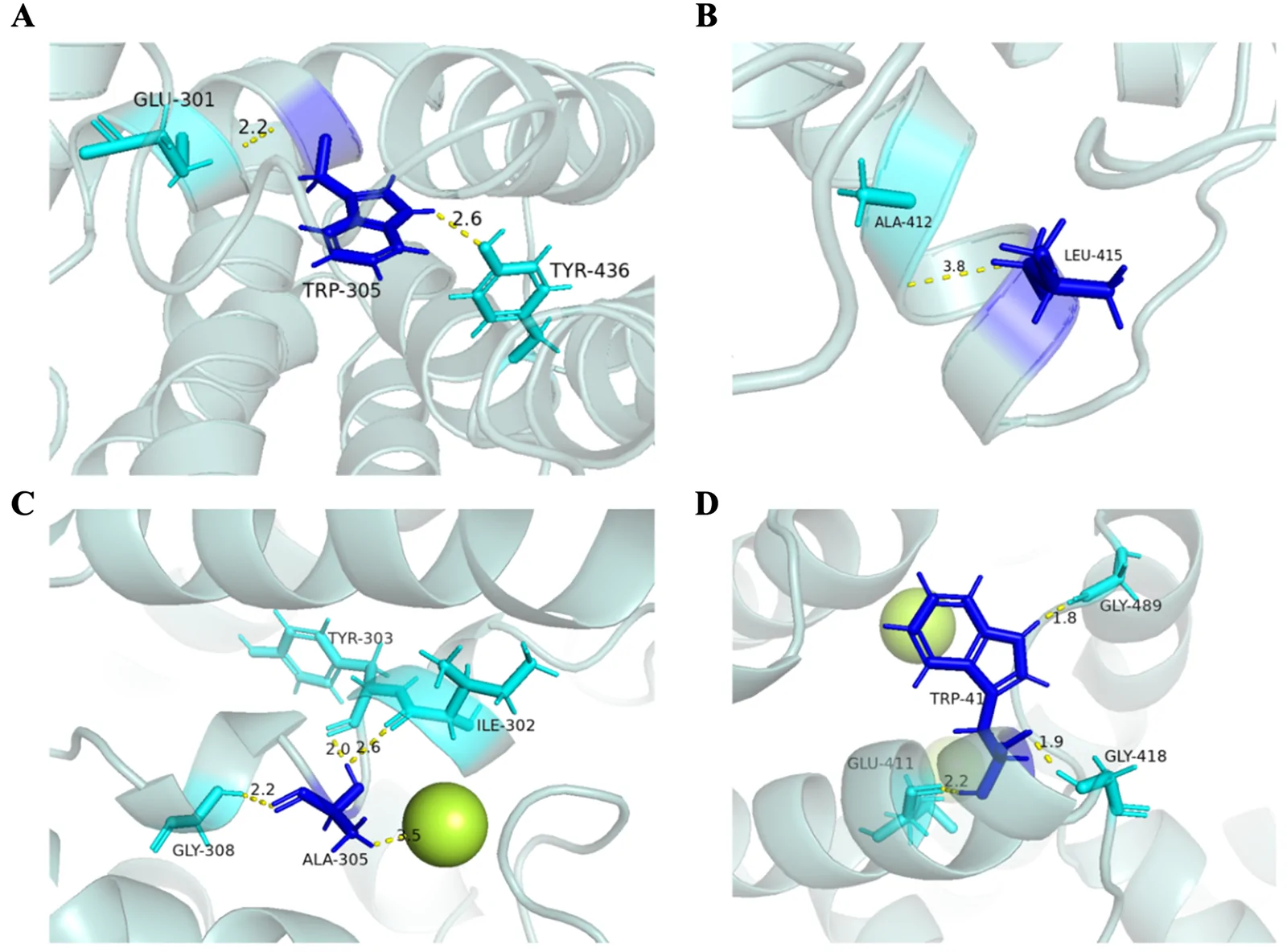
Structure comparison of CiGASlo variant at site 305 and 415 with wild-type. **A**: W305; **B**: L415; **C**: W305A; **D**: L415W

For the W305A/L415W double variant, MD simulations suggested reduced local fluctuation around selected pocket-adjacent regions compared with the wild-type enzyme (Fig. 5B). The average radius of gyration of W305A/L415W over the equilibrated trajectory was close to that of the wild type, and the small numerical difference was not interpreted as evidence for meaningful global compaction. Instead, the structural effect of W305A/L415W was mainly reflected in local interaction changes. Hydrogen-bond analysis showed that W305A/L415W displayed a slightly higher average number of intramolecular hydrogen bonds than the wild type, with 2,558 versus 2,553 average hydrogen bonds over the trajectory (Fig. 5D). Because this difference is small, it was not used alone as proof of enhanced thermostability. However, local interaction analysis indicated that W305A may introduce new contacts involving Ala305–Tyr303 and Ala305–Gly308, whereas L415W may form additional local interactions with Gly418, Glu411, and Gly489 (Fig. 6). These local interactions may contribute to the reduced flexibility of specific pocket-adjacent regions and help explain the relatively favorable residual activity of W305A/L415W at 30 °C.

Together, the MD simulations suggest that different mutations modulate CiGASlo through distinct local structural effects. Mutations at conserved metal-binding residues, such as D333A, D337A, and D477A, may reduce local flexibility but impair catalytic activity by disturbing Mg^2+^ coordination. R488A may improve apparent catalytic efficiency by altering the local environment near the substrate-access or product-release region. W305A/L415W may enhance operational stability under fermentation-relevant temperature conditions by modifying local interaction patterns around residues 305 and 415. These MD-based interpretations are consistent with the biochemical trends, but they should be regarded as structural hypotheses. High-resolution experimental structures of CiGASlo and its engineered variants, ideally in complex with FPP or substrate analogs, will be required to validate the detailed conformational effects of these mutations.

### Fermentation optimization for β-elemene producing *Saccharomyces cerevisiae*

Although R488A exhibited the highest apparent catalytic efficiency in vitro, the W305A/L415W variant was selected for yeast fermentation because β-elemene accumulation in vivo depends on cumulative enzyme performance during long-term cultivation rather than on purified-enzyme catalytic efficiency alone. As shown above, W305A/L415W displayed the highest apparent specific activity, the highest apparent *V*max, the highest kcat, and favorable residual activity under fermentation-relevant temperature conditions. Therefore, this variant was considered suitable for validating whether enzyme-level improvement could be translated into enhanced β-elemene production in a microbial cell factory.

**Fig. 7:**
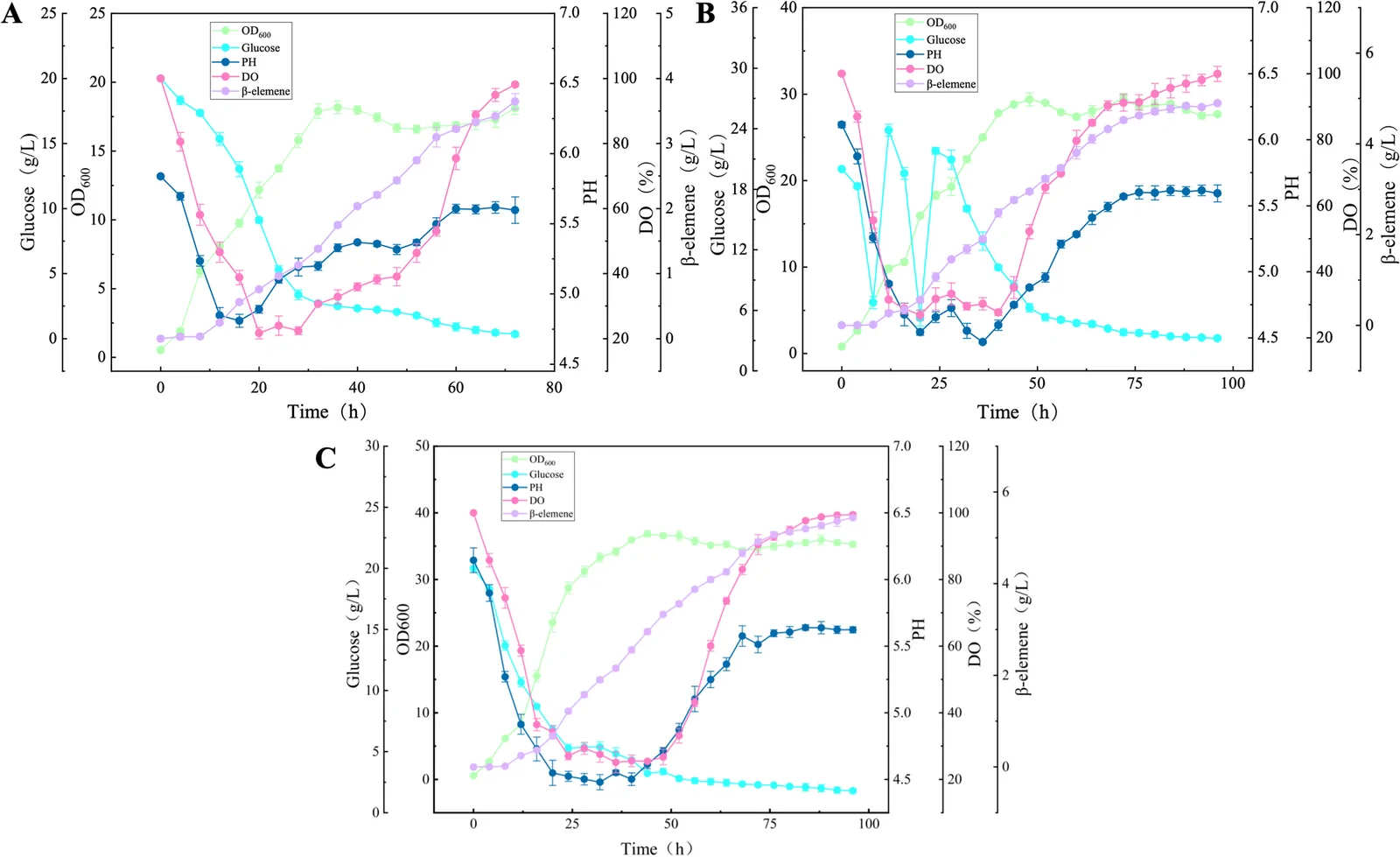
Fermentation curve of *Saccharomyces cerevisiae* with CiGASlo-W305A/L415W. **A**: batch fermentation; **B**: fed-batch fermentation; **C**: Continuous fed-batch fermentation

To evaluate the production performance of the W305A/L415W-containing strain, fermentation experiments were conducted in a 5 L glass bioreactor at 30 °C with pH controlled at 6.5–7.0. Three representative fermentation strategies were compared: batch fermentation, fed-batch fermentation, and continuous-feeding fermentation. These strategies were designed to assess how nutrient-supply mode affected cell growth and β-elemene accumulation in the recombinant S. cerevisiae strain B2-11. In batch fermentation, strain B2-11 reached a final OD_600_ of 18.16 and produced 3.65 g·L^-1^ β-elemene (Fig. 7A). This result demonstrated that the W305A/L415W-containing strain was capable of accumulating β-elemene at gram-per-liter scale under simple batch conditions. However, the limited nutrient availability in batch fermentation likely restricted further biomass formation and product accumulation.

Fed-batch fermentation further improved both cell growth and β-elemene production (Fig. 7B). The final OD_600_ increased to 29.49, representing a 62.39% increase compared with batch fermentation. Correspondingly, the β-elemene titer reached 4.89 g·L^-1^, which was 34.19% higher than that obtained in batch fermentation. The improved performance can be attributed to additional nutrient supplementation during cultivation, which alleviated nutrient limitation and supported higher cell density and product accumulation. Among the three strategies tested, continuous-feeding fermentation showed the best performance (Fig. 7C). Under this strategy, nutrients were supplied more continuously, enabling more stable cell growth during the production phase. The final OD_600_ reached 36.86, which was 24.99% higher than that achieved in fed-batch fermentation. β-Elemene production further increased to 5.44 g·L^-1^, representing an 11.25% improvement over fed-batch fermentation and a 49.04% improvement over batch fermentation. This titer was also 15.7% higher than the previously reported β-elemene production benchmark of 4.70 g·L^-1^ in Ogataea polymorpha [43], highlighting the potential of the engineered CiGASlo variant and optimized yeast fermentation strategy for high-level β-elemene biosynthesis.

These fermentation results indicate that nutrient-supply strategy strongly affects β-elemene production in *S. cerevisiae*. Compared with batch fermentation, fed-batch and continuous-feeding strategies supported higher biomass accumulation and improved product titer, suggesting that sustained nutrient availability is important for maintaining precursor supply and cumulative terpene production. The superior performance of continuous-feeding fermentation further indicates that controlled nutrient delivery can improve the balance between cell growth and β-elemene biosynthesis.

It should be noted that the present fermentation comparison focused on three representative feeding strategies rather than exhaustive statistical optimization of all process variables. Therefore, further improvement will require systematic process development. In future work, Design of Experiment (DoE)-based approaches, such as Plackett–Burman screening followed by response-surface methodology or Box–Behnken design, could be used to identify and optimize key parameters, including glucose feeding rate, yeast extract feeding rate, pH, dissolved oxygen, agitation speed, fermentation temperature, and feeding duration. Such an approach would allow quantitative evaluation of parameter interactions and could further improve β-elemene titer, yield, and productivity.

## Conclusion

In this study, CiGASlo was engineered using a semi-rational strategy that combined structural modeling, molecular docking, focused mutagenesis, biochemical characterization, MD-assisted analysis, and yeast fermentation validation. The computational workflow was used to prioritize substrate-proximal and structurally tolerated residues, while catalytic improvements were determined experimentally. Several variants showed improved apparent activity or kinetic performance. Semi-rational engineering identified several CiGASlo variants with distinct performance advantages. L415W provided the strongest combination of substrate affinity, catalytic efficiency, and a favorable numerical thermal profile, whereas W305A/L415W showed the highest specific activity under the screening conditions and supported 5.44 g L^-1^ (−)-β-elemene production in the engineered yeast strain under continuous feeding. Because L415W was not tested in a matched fermentation, the present data establish the feasibility, but not the relative superiority, of W305A/L415W in vivo. Future work should focus on high-resolution structural determination, broader mutational exploration, pathway balancing, DoE-guided fermentation optimization, and scale-up to further improve β-elemene titer, yield, and productivity.

## Supplementary Information

Below is the link to the electronic supplementary material.

Supplementary Material 1

## Data Availability

No datasets were generated or analysed during the current study.

## Abbreviations

CiGASlo: Germacrene A synthase from Cichorium intybus
CRISPR: Clustered regularly interspaced short palindromic repeats
EA: Enzyme activity
FPP: Farnesyl diphosphate
GAS: Germacrene A synthase
GC: Gas chromatography
MD: Molecular dynamics
MVA: Mevalonate
NPT: Constant number of particles, pressure, and temperature
NVT: Constant number of particles, volume, and temperature
Rg: Radius of gyration
RMSD: Root mean square deviation
RMSF: Root mean square fluctuation
sgRNA: Single-guide RNA
TPS: Terpene synthase
