# Supplementary material for: Semi-rational design and engineering of chicory-derived germacrene A synthase for enhanced (−)-β-elemene production in Saccharomyces cerevisiae
Source: BMC Biotechnol. 2026 Aug 12;26:132. doi: 10.1186/s12896-026-01212-3 (PMC13746407; doi:10.1186/s12896-026-01212-3)
Supplement: Supplementary file 1 [file 12896_2026_1212_MOESM1_ESM.docx]

**Supporting information**

**Semi-rational Design and Engineering of Chicory-derived Germacrene A Synthase for Enhanced (−)-β-Elemene Production in *Saccharomyces cerevisiae***

Da-Ming Wang^a,*^, Si-Jia Song^a^, Kang-Yue Ren^a^, Lei Sun^b^, Yong-Kun Du^a^, Hao Guo^a^, Tian Xie^a,**^

^a^ Key Laboratory of Elemene Class Anti-Cancer Medicines, School of Pharmacy, Hangzhou Normal University, Hangzhou, 311121, PR China

^b^ School of Food and Biological Engineering, Jiangsu University, Zhenjiang, 212013, P. R. China

Corresponding authors

* Corresponding author. Key Laboratory of Elemene Class Anti-Cancer Medicines, School of Pharmacy, Hangzhou Normal University, Hangzhou, 311121, P. R. China.

** Corresponding author. Key Laboratory of Elemene Class Anti-Cancer Medicines, School of Pharmacy, Hangzhou Normal University, Hangzhou, 311121, P. R. China.

E-mail addresses: wdm@hznu.edu.cn (D.-M. Wang), tianxie@hznu.edu.cn (T. Xie)

**Table S1.** Reported microbial production of germacrene A/β-elemene using different GAS enzymes.

| **Expression host** | **GSA enzyme** | **Shake-flask titer（mg/L）** | **Fed-batch titer（g/L）** | **Productivity（mg/L/h）** | **References** |
| --- | --- | --- | --- | --- | --- |
| *E. coli* | LTC2（*Lactuca sativa*） | 126 | - | 7.02 | Chen et al., 2022 |
| *E. coli* | LTC2（*Lactuca sativa*） | 364 | - | - | Li et al., 2022 |
| *S. cereviside* | LTC2（*Lactuca sativa*） | 190 | - | 2.65 | Hu et al., 2017 |
| S. *Cereviside* | AvGAS（*Anabaena variabilis*） | 310 | - | 4.30 | Zhang et al., 2021 |
| *S. Cerevisiae* | CiGASlo（*Cichorium intybus*） | 1152 |  |  | Hu et al., 2024 |
| *O. polymorpha* | LTC2（*Lactuca sativa*） | 509 | 4.70 | 17.80 | Ye et al., 2023 |
| *P. pastoris* | AvGAS（*Anabaena variabilis*） | 335 | 1.90 | 19.79 | Cheng et al., 2023 |
| *Y. lipolytica* | DlGAS（*Daldinia loculata*） | 2794 | 39.00 | 162.50 | Liu et al., 2023 |
| *Y. lipolytica* | LTC2（*Lactuca sativa*） | 785 | 5.08 | 26.46 | Li et al., 2023 |

**Note:** “–” indicates that the value was not reported. GAS, germacrene A synthase. Direct comparison should be interpreted with caution because some studies reported germacrene A, whereas others reported β-elemene after thermal or chemical conversion.

**Table S2.** Related data of Germacrene A synthase reported in literature.

| **GAS** | **Source** | **Number of**  **amino acids** | ***K*_m_**  **(µM)** | ***K*_cat_**  **(s^-1^)** | **pH**  **(-)** | **Genebank ID** | **References** |
| --- | --- | --- | --- | --- | --- | --- | --- |
| LsLTC1 | *Lactuca sativa* | 559 | / | / | 7 | AF489964 | Bennett et al., 2002 |
| LsLTC2 | *Lactuca sativa* | 557 | 10.9 | 0.28 | 7 | AF489965 | Bennett et al., 2002; Nguyen et al., 2016 |
| CiGASsh | *Cichorium intybus* L | 558 | 3.2 | / | 7 | AF498000 | Bouwmeester et al., 2002 |
| CiGASlo | *Cichorium intybus* L. | 583 | 6.9 | / | 6.8 | AF497999 | Bouwmeester et al., 2002 |
| BsGAS1 | *Barnadesia spinosa* | 580 | 7.8 | 0.13 | / | KM066976 | Nguyen et al., 2016 |
| BsGAS2 | *Barnadesia spinosa* | 554 | 14.8 | 0.28 | / | KM066977 | Nguyen et al., 2016 |
| AaGAS | *Artemisia annua* | 562 | 10.8 | 0.25 | / | DQ447636 | Bertea et al., 2006; Nguyen et al., 2016 |
| ScGAS | *Solidago canadensis* | 549 | 2.5 | / | 7 | AJ304452 | Prosser et al., 2002 |
| IdGAS | *Ixeris dentata* | 584 | 11 | 0.016 | 7.5~8 | AY082672 | Chang et al., 2005 |
| IdGASm | *Ixeris dentata* | / | 25.1 | 0.044 | / | / | Chang et al., 2005 |
| AmGAS | *Achillea millefolium* | 559 | / | / | / | KC145534 | Pazouki et al., 2015 |
| HaGAS1 | *Helianthus annuus* | 559 | 0.82 | / | 7.7 | DQ016667 | Göpfert et al., 2009 |
| HaGAS2 | *Helianthus annuus* | 559 | 0.74 |  | 7.5 | EU327785 | Göpfert et al., 2009 |
| TcGAS | *Tanacetum cinerariifolium* | 561 | / | / | / | KC441526 | Ramirez et al., 2013 |
| PcGAS | *Pogostemon cablin* | 554 | 4.45 | 0.43 × 10^-3^ | 7~7.5 | AY508728 | Deguerry et al., 2006 |
| CcGAS | *Cynara cardunculus* *var. scolymus* L. | 559 | / | / | / | JN383985 | Menin et al., 2012 |
| AcGAS | *Aralia cordata* | 555 | / | / | / | BBE38101 | Shindo et al., 2018 |
| AvGAS | *Vitis vinifera* | 555 | / | / | / | ADR66821 | Martin et al., 2010 |
| ToGAS1 | *Taraxacum officinale* | 559 | / | / | / | KT898039 | Huber et al., 2016 |
| ToGAS2 | *Taraxacum officinale* | 583 | / | / | / | KT898040 | Huber et al., 2016 |
| TpGAS | *Tanacetum parthenium* L. | 559 | / | / | / | JF819848 | Majdi et al., 2011 |
| ApGAS | *Artemisia pallens* | 559 | / | / | / | OR631198 | Kiran et al., 2024 |
| ShGAS | *Solanum habrochaites* | 540 | / | / | / | G8H5N2 | Bleeker et al., 2011 |
| CfGAS | *Chamaecyparis formosensis* | 589 | / | / | / | MG930776 | Ma et al., 2021 |

Note: “/” indicates that the value was not reported. GAS, germacrene A synthase. The entry originally labeled as “AvGAS” from Vitis vinifera was revised to “VvGAS” to match the source organism.

**Table S3.** Primers used in this study.

| **Primer** | **Sequence（5’—3’）** |
| --- | --- |
| P44W-F | GTTCGTtggCTGGCAAACTTTCCGCCGTCAAT |
| P44W-R | TTTGCCAGccaACGAACCGGTTCAGGGGTCGG |
| W305A-F | TATCTGgcaATTCTGGGTCTGTATTTTGAACCG |
| W305A-R | CCCAGAATtgcCAGATAAATTTCCGGAACGCG |
| L309M-F | GTGGATTCTGGGTatgTATTTTGAACCGTATTATTCTCGTGC |
| L309M-R | AcatACCCAGAATCCACAGATAAATTTCCGGA |
| T326A-F | CGACCAAAATTgcaCTGTTTCTGGTTGTTCTGGATGAT |
| T326A-R | CAGtgcAATTTTGGTCGCAATAATACGCGCAC |
| T326Y-F | CGACCAAAATTtatCTGTTTCTGGTTGTTCTGGATGAT |
| T326Y-R | CAGataAATTTTGGTCGCAATAATACGCGCAC |
| D333A-F | GTTGTTCTGgcaGATACTTATGATGCATATGCAACCATT |
| D333A-R | GTATCtgcCAGAACAACCAGAAACAGGGTAATT |
| D333H-F | CTGGTTGTTCTGcatGATACTTATGATGCATATGCAACCATT |
| D333H-R | TCatgCAGAACAACCAGAAACAGGGTAATTTT |
| D337A-F | ATgcaGCATATGCAACCATTGATGAAATTCGT |
| D337A-R | GGTTGCATATGCtgcATAAGTATCATCCAGAACAACCAGAAA |
| D337H-F | ATcatGCATATGCAACCATTGATGAAATTCGT |
| D337H-R | GGTTGCATATGCatgATAAGTATCATCCAGAACAACCAGAAA |
| Y408F-F | ACGTGGCtttCTGGAAGAAGCAGAATGGCTGC |
| Y408F-R | CTTCCAGaaaGCCACGTGCAATTTCCTGAAAT |
| L415W-F | GAATGGtggCATAATGGTTATGTTGCAACATTTCC |
| L415W-R | CCATTATGccaCCATTCTGCTTCTTCCAGATAGC |
| A421P-F | ATGTTccgACATTTCCGGAATATATGAAAAATGG |
| A421P-R | CGGAAATGTcggAACATAACCATTATGCAGCCATTC |
| N429Y-F | tatGGTCTGATTACCTCAGCATATAACGTTAT |
| N429Y-R | GAGGTAATCAGACCataTTTCATATATTCCGGAAATGTTGC |
| A435G-F | GTCTGATTACCTCAggtTATAACGTTATTAGCAAAAGCGCA |
| A435G-R | accTGAGGTAATCAGACCATTTTTCATATATT |
| S473A-F | ACTGATTgcaCGTCTGCAGGATGATGTTATGACA |
| S473A-R | GCAGACGtgcAATCAGTTCTGAGGCTTTCAGAATT |
| R474L-F | TGATTTCCctgCTGCAGGATGATGTTATGACATTTC |
| R474L-R | CTGCAGcagGGAAATCAGTTCTGAGGCTTTCA |
| D477A-F | TCTGCAGgcaGATGTTATGACATTTCAGTTTGAACG |
| D477A-R | TAACATCtgcCTGCAGACGGGAAATCAGTTCT |
| D477H-F | GTCTGCAGcatGATGTTATGACATTTCAGTTTGAACG |
| D477H-R | AACATCatgCTGCAGACGGGAAATCAGTTCTG |
| D478S-F | GCAGGATagcGTTATGACATTTCAGTTTGAACGCA |
| D478S-R | TCATAACgctATCCTGCAGACGGGAAATCAGT |
| E485A-F | TCAGTTTgcaCGCAAACGTGGTCAGAGCGCAA |
| E485A-R | GTTTGCGtgcAAACTGAAATGTCATAACATCATCCTG |
| E485Y-F | TtatCGCAAACGTGGTCAGAGCGCAACAGGTG |
| E485Y-R | GACCACGTTTGCGataAAACTGAAATGTCATAACATCATCCTG |
| R488A-F | ACGCAAAgcaGGTCAGAGCGCAACAGGTGTTG |
| R488A-R | TCTGACCtgcTTTGCGTTCAAACTGAAATGTCA |
| R488L-F | ACGCAAActgGGTCAGAGCGCAACAGGTGTTG |
| R488L-R | TCTGACCcagTTTGCGTTCAAACTGAAATGTCA |
| Q490W-F | ACGCAAACGTGGTtggAGCGCAACAGGTGTTGATGC |
| Q490W-R | TccaACCACGTTTGCGTTCAAACTGAAATGTC |
| S491Y-F | TGGTCAGtatGCAACAGGTGTTGATGCGTATATT |
| S491Y-R | CTGTTGCataCTGACCACGTTTGCGTTCAAAC |
| A492P-F | AGAGCccgACAGGTGTTGATGCGTATATTAAAGAA |
| A492P-R | AACACCTGTcggGCTCTGACCACGTTTGCGTT |
| T493S-F | AagcGGTGTTGATGCGTATATTAAAGAATATAA |
| T493S-R | ACGCATCAACACCgctTGCGCTCTGACCACGTTTG |
| Y553F-F | GATTGATGTGGTCtttAAATTTGATGATGGCTTTACGTTTC |
| Y553F-R | TaaaGACCACATCAATCATACGTGCCAGATTC |
| pET28a-*CiGASlo*-CF1-F | ACCATGCCGTCAATTTGGGCAGATCGTTTTATT |
| pET28a-*CiGASlo*-CF1-R | CCAAATTGACGGCATGGTATATCTCCTTCTTAAAGTTAAAC |
| pET28a-*CiGASlo*-CF2-F | GTTCGTCCTCTGGCAAAC |
| pET28a-*CiGASlo*-CF3-F | CAGCCGACCCCTGAAC |
| pET28a-*CiGASlo*-CF4-F | CCTCTGTCAGTTCAGCCG |
| pET28a-*CiGASlo*-CF5-F | CTGACCAGCCCTCGT |
| pET28a-*CiGASlo*-CF-R | CATGGTATATCTCCTTCTTAAAGTTA |

**Table S4** Predicted ΔΔG values of CiGASlo mutants.

| Mutant | L309 | T326 | D333 | D337 | Y408 | L415 | A421 | N429 | A435 | R474 | D477 | D478 | E485 | R488 | Q490 | S491 | A492 | T493 | Y553 |
| --- | --- | --- | --- | --- | --- | --- | --- | --- | --- | --- | --- | --- | --- | --- | --- | --- | --- | --- | --- |
| A | -3.2 | -0.7 | -0.3 | -0.3 | -3.0 | -0.7 | 0.0 | -0.6 | 0.0 | -0.9 | -0.4 | -1.7 | -1.4 | -1.2 | -0.7 | 0.0 | 0.0 | -2.2 | -3.6 |
| C | -2.8 | 0.0 | -0.9 | -0.5 | -1.8 | -0.3 | 0.2 | -0.9 | 0.7 | -1.2 | -0.6 | -2.4 | -1.8 | -1.5 | -0.2 | 0.2 | -0.3 | -1.4 | -1.6 |
| D | -4.9 | -4.9 | 0.0 | 0.0 | -3.3 | -1.7 | -1.2 | -1.9 | -0.7 | -2.7 | 0.0 | 0.0 | -1.2 | -1.8 | -0.5 | -0.3 | -1.1 | -4.2 | -4.5 |
| E | -3.8 | -2.5 | -0.3 | -0.3 | -3.4 | -0.9 | -0.6 | -1.4 | -0.5 | -3.2 | -0.7 | -2.8 | 0.0 | -1.4 | 0.0 | 0.3 | -1.5 | -3.7 | -4.4 |
| F | -0.5 | 0.9 | 0.7 | 0.6 | -0.9 | 0.2 | 0.5 | 1.4 | 1.2 | -0.2 | 1.5 | -1.9 | -0.6 | -0.8 | 0.1 | 0.3 | -0.2 | -1.4 | -0.9 |
| G | -5.2 | -3.2 | -1.5 | -1.1 | -4.1 | -1.7 | -1.1 | -2.4 | 1.2 | -2.4 | -1.5 | -3.2 | -2.2 | -1.9 | -1.2 | -0.2 | -0.7 | -3.3 | -4.5 |
| H | -4.4 | -2.5 | -0.5 | -0.5 | -2.9 | -0.8 | -0.5 | -0.6 | -0.8 | -2.3 | -0.3 | -3.5 | -2.1 | -1.5 | 0.4 | 0.1 | -0.6 | -3.0 | -3.0 |
| I | -1.3 | 1.5 | 0.6 | 0.3 | -2.0 | -0.1 | 0.2 | 0.5 | 0.7 | 0.0 | 0.7 | -1.6 | -0.3 | -0.1 | -0.2 | 0.5 | -0.2 | -1.8 | -3.2 |
| K | -3.7 | -3.7 | -0.8 | -0.8 | -2.9 | 0.0 | -0.1 | -1.2 | -0.6 | -2.2 | -0.5 | -3.9 | -1.7 | -1.4 | 0.0 | 0.2 | -0.8 | -2.5 | -3.3 |
| L | 0.0 | 1.4 | 1.0 | 0.6 | -0.5 | 0.0 | 1.3 | 1.0 | 1.5 | 0.2 | 1.3 | -2.0 | 0.2 | 0.2 | 0.5 | 0.5 | -0.3 | -2.0 | -1.7 |
| M | -0.6 | 0.5 | 0.2 | 0.1 | -1.4 | -0.3 | 0.2 | 0.1 | 0.5 | -0.4 | 0.2 | -1.9 | -0.4 | -0.3 | -0.3 | 0.1 | -0.4 | -2.7 | -2.4 |
| N | -5.2 | -1.8 | -0.4 | -0.6 | -2.4 | -0.9 | -0.7 | 0.0 | 0.0 | -2.3 | 0.1 | -2.7 | -1.5 | -1.8 | -0.3 | 0.1 | -1.0 | -2.7 | -3.3 |
| P | -5.0 | -4.9 | -1.6 | -0.8 | -5.1 | -2.4 | 1.4 | -2.3 | -0.1 | -3.0 | -2.1 | -3.6 | -2.0 | -2.1 | -2.0 | 0.5 | -0.6 | -4.6 | -5.2 |
| Q | -4.3 | -3.9 | -0.4 | -0.3 | -3.9 | -0.4 | -0.6 | -0.7 | -1.1 | -1.9 | -0.6 | -3.5 | -0.8 | -0.9 | 0.0 | 0.1 | -1.1 | -3.5 | -4.2 |
| R | -5.2 | -3.8 | -0.7 | -0.6 | -3.4 | -0.3 | -0.4 | -1.6 | -0.2 | 0.0 | -1.1 | -3.5 | -1.3 | 0.0 | -0.4 | 0.0 | -1.0 | -2.8 | -4.4 |
| S | -3.7 | -1.1 | -0.2 | -0.2 | -3.8 | -0.7 | -0.4 | -0.4 | -0.4 | -1.4 | -0.3 | -1.5 | -1.0 | -1.2 | -0.8 | 0.0 | -0.8 | 0.1 | -3.8 |
| T | -2.3 | 0.0 | 0.0 | -0.2 | -3.2 | -0.5 | -0.3 | 0.0 | 0.5 | -1.4 | 0.1 | -2.7 | -1.0 | -1.4 | -0.5 | 0.1 | -0.8 | 0.0 | -3.3 |
| V | -0.8 | 0.4 | 0.4 | 0.0 | -2.1 | -0.5 | 0.1 | 0.7 | 0.8 | -1.1 | 0.4 | -2.1 | -1.2 | -1.2 | 0.0 | 0.9 | -0.5 | -1.6 | -2.6 |
| W | -1.7 | -0.1 | -0.2 | 0.0 | -1.5 | -0.3 | 0.8 | 0.5 | 1.3 | -1.1 | 0.4 | -2.2 | -0.7 | -0.7 | 0.4 | 0.5 | 0.3 | -2.1 | -3.0 |
| Y | -3.8 | -1.2 | -0.9 | -0.2 | 0.0 | 0.2 | 0.4 | -0.2 | 1.1 | -2.2 | -0.4 | -3.1 | -1.3 | -1.5 | -0.4 | 0.4 | -0.2 | -3.4 | 0.0 |

# Supplementary Figures

**Figure S1.** Phylogenetic analysis of germacrene A synthase.

**Figure S2.** The overlay of the X-ray structure of the crystal structure of (PDB: 5EAU) and the simulated model of CiGASlo. Silver, LcGAS, Blue:CIGASlo.

**Figure S3.** Docking results of substrate FPP with CiGASlo.

**Figures S4.** Chiral structure identification diagram of β-elemene.
